# *FCGR2A*-131R Is Associated with Lupus Nephritis Rather than Non-Lupus Nephritis SLE in an Indigenous African Caribbean Population

**DOI:** 10.3390/cimb47070490

**Published:** 2025-06-26

**Authors:** Fatima Radouani, Christophe Deligny, Raymond Cesaire, Maryvonne Dueymes, Georges Dos Santos

**Affiliations:** 1 Laboratory of Immunology, University Hospital of Martinique (CHU Martinique), 97200 Fort de France, France; 2 Cardiovascular Research Team (UR5_3 PC2E), University of the French West Indies (Université des Antilles), 97200 Fort de France, France; 3 Research Department, University Hospital of Martinique (CHU Martinique), 97200 Fort de France, France; 4 Department of Internal Medicine, University Hospital of Martinique (CHU Martinique), 97200 Fort de France, France; 5 Department of Virology, University Hospital of Martinique (CHU Martinique), 97200 Fort de France, France; 6 Department of Virology, University Hospital of Guadeloupe (CHU Guadeloupe), 97159 Pointe-à-Pitre, France; 7 B lymphocytes, Autoimmunity and Immunotherapy (LBAI), Mixte Research Unit 1227 (UMR 1227 INSERM), University of Western Brittany, 29200 Brest, France; 8 Laboratory of Immunology, University Hospital of Brest, 29200 Brest, France

**Keywords:** FcγRs polymorphisms, systemic lupus erythematosus, African Caribbean, lupus nephritis

## Abstract

Fc gamma receptors (FcγRs) control humoral and cellular immune responses and maintain the immune system balance. Functional polymorphisms of FcγRs, whose prevalence was dependent on ethnic origin, were found to be associated with systemic lupus erythematosus (SLE) or kidney injuries in several ethnic groups. We aimed at investigating the association between the functional single-nucleotide polymorphisms (SNPs) of FcγRIIa-H131R (rs1801274), FcγRIIb-I232T (rs1050501), FcγRIIIa-V158F (rs396991) and FcγRIIIb variants (NA1 and NA2) and lupus erythematosus systemic in an indigenous African Caribbean population. We compared the frequencies of the functional SNPs of *FCGR2A* (FcγRIIa-H131R, rs1801274), *FCGR2B* (FcγRIIb-I232T, rs1050501), *FCGR3A* (FcγRIIIa-V158F, rs396991) and *FCGR3B* variants (FcγRIIIb NA1 and NA2) between lupus and healthy controls in an indigenous African Caribbean population. We highlighted an association between the *FCGR3B*-NA1/NA1 and *FCGR3A*-158F alleles and systemic lupus erythematosus, in addition to an association between *FCGR2A*-131R and lupus nephritis. Furthermore, an increase in the 131R-158V haplotype in lupus nephritis (30.4%) vs. lupus non-nephritis (15.8%) was noticed. Surprisingly, in spite of the high frequency of the *FCGR2B*-232T allele in our population, our study did not highlight any association of this allele either with SLE or lupus nephritis (a severe and frequent form of SLE). CD72-Hap1, which has been shown to confer resistance to SLE against T232 allele, was not enhanced in the control group. Our results emphasize an association between *FCGR2A*-131R and lupus nephritis with a distinctive *FCGR* polymorphism distribution in an indigenous African Caribbean population, confirming the important variation in the *FCGR* locus depending on ethnic origin.

## 1. Introduction

Systemic lupus erythematosus (SLE) is a prototype of autoimmune disease characterized by high level of autoantibody production, leading to multiple organ inflammation.

With an incidence of 5.14 per 100,000 and a prevalence of 43.7 per 100,000, approximately 3.4 million people worldwide have SLE, 40% of whom develop lupus nephritis (LN) [1,2]. In addition to a gender-dependent prevalence (90% of patients with SLE are female) [3], ethnicity is also an important key factor of high frequency and severity. In fact, SLE is more prevalent in non-Europeans, such as Asians and African Americans [4,5,6].

Fc gamma receptors (FcγRs) are cell surface glycoproteins that bind to the Fc fragment of IgG and are crucial for regulating effector cells and linking the humoral to the cellular immune response [7]. Multiple FcγRs, which differ in their ligand affinities, cellular distributions and effector functions, constitute a clustered gene family (*FCGRs*) on chromosome 1q21-24. Interestingly this region is strongly associated with SLE [8].

In humans, FcγRs are classified into three main types, each containing multiple distinct genes and alternative splicing variants associated with SLE [8]. This classification is determined according to their structure and function: (i) FcγRI (CD64 in the classification of the cluster of differentiation): It has a high affinity to IgG, and it is expressed on monocytes, macrophages, dendritic cells (DCs), neutrophils [9]. (ii) The low-affinity FcγRII (CD32) has three subtypes: FcγRIIa is expressed on monocytes, macrophages, DCs, neutrophils, and platelets; FcγRIIb, the only inhibitory FcγR, is expressed on a variety of immune cells, including B cells, monocytes, DCs, macrophages, neutrophils, basophils and mast cell; and FcγRIIc is expressed on B lymphocytes and natural killer (NK). Finally, (iii) FcγRIII (CD16), the other low-affinity FcγRs, has two subtypes: FcγRIIIa and FcγRIIIb.

In *FCGR2A*, a single base change from guanine (G) to adenine (A) at nucleotide 519 (rs1801274) results in a substitution of arginine (R) with histidine (H) at the 131 position of the second extracellular Ig-like domain of FcγRIIa [10]. This substitution increases the affinity of the receptor to IgG2 [11,12], affecting the phagocytosis mediated by this IgG [13]. In *FCGR2B*, there is a non-synonymous substitution of thymine (T) to cytosine (C). This single-nucleotide polymorphism (SNP) (rs1050501) encodes an isoleucine (I)-to-threonine (T) substitution at position 232 in the transmembrane domain of FcγIIb. This substitution leads to an impairment of the inhibitory function of the receptor through its exclusion from lipid rafts [14,15].

In *FCGR3A*, a substitution of T to G at nucleotide 559 (rs396991) changes the phenylalanine (F) to valine at position 158 of FcγRIIIa. The FcγRIIIa-158V variants display a relatively higher affinity for IgG1 and IgG3 compared to the FcγIIIa-158F one [16].

Finally, in *FCGR3B*, two serologically defined haplotypes, NA1 and NA2, have been described. When compared to homozygous NA2, homozygous NA1 has been described to exhibit a higher phagocytic capacity due to its efficacy in binding immune complexes containing IgG1 and IgG3 [17].

Frequencies of these functional polymorphisms depend on ethnic origin and are associated with SLE and/or LN in several ethnic groups, including Asians, Europeans, Africans, and African Americans [18]. In spite of its strong association with SLE and its severity, the risk of FcγRIIb-232T conferring SLE is thought to decrease in the presence of a specific haplotype (Hap) of CD72 [19]. CD72 is a 45 KDa type II transmembrane protein containing a C-type Lectin domain. It is expressed in most developmental stages of B cells except plasma cells. CD72 plays a negative regulatory role in B cell receptor (BCR) signaling after its stimulation with IgM or IgG [20,21]. Genotypically, two major haplotypes (Hap) 1 or 2 of CD72 exist, containing, respectively, one or two repeats of 13 nucleotides in intron 8. Hap2 provokes apoptosis in B cells by increasing an alternative splicing, resulting in a longer protein and its sequestration in reticulum endoplasmic [22]. Thus, Hap2 reduces the risk of developing SLE [19].

In Martinique, a Caribbean Island, where the population has mainly an African Caribbean origin [23], SLE is potentially severe, with an increased incidence of LN [24]. In addition, SLE is one of the most frequent causes of dialysis in this island [25].

In this study, we have determined for the first time, the frequencies of FcγRIIa-H131R, FcγRIIb-I232T, FcγRIIIa-F158V, and FcγRIIIb-NA1/NA2 polymorphisms and their association with SLE and LN in 126 patients with SLE including 58 patients with LN and 120 unrelated controls from this indigenous African Caribbean population. Furthermore, we have explored for the first time CD72 polymorphism in an African descendent population to explain the exceptionally high frequency of FcγRIIb 232T/T in this indigenous African Caribbean population.

## 2. Materials and Methods

### 2.1. Ethics Statement

Ethical approval for the study was given by the “Comité de protection des personnes Sud Ouest et Outremer III” under number 2013-A01610-45. Written informed consent was obtained from all participants.

### 2.2. Study Subjects

Anticoagulated peripheral blood was obtained from 120 healthy volunteers from Martinique and from the 126 patients included because they fulfilled the revised 1997 American College of Rheumatology (ACR) criteria for SLE [26]. We excluded children, childbearing patients and those without social security. Patients with SLE were recruited at the Internal Medicine Department of the University Hospital of Martinique at Fort de France. All donors self-reported as originating from Martinique and living in Martinique, and provided informed written consent.

### 2.3. Genotyping of FCGR2A, FCGR2B and FCGR3A

Genomic DNA was purified from peripheral blood mononuclear cells of healthy subjects and patients using QIAamp DNA blood Maxi kit (Qiagen, Hilden, Germany). For PCRs, the following primers were used: 5′-CTGAGACTGAAAAACCCTTGGAATC-3′ (forward) and 5′-GCTTGTGGGATGGAGAAGGTGGGATCCAAA-3′ (reverse) for *FCGR2A* [27]. 5′-CCTGCCTGCTCACAAATGTA-3′ (forward) and 5′-CTGAAATCCGCTTTTTCCTG-3′ (reverse) for *FCGR2B* gene [28,29]. 5′-TATTTACAGAATGGCAAAGG-3′ (forward) and 5′-GTGATGGTGATGTTCACAGT-3′ (reverse) for *FCGR3A* [30]. PCRs were run in 50 µL containing 100 ng of DNA, 2.5 mM of MgCl_2_, 200 µM of dNTPs, 0.4 µM of each primer and 1.25 unit of AmpliTaq Gold 360 DNA Polymerase (Thermo Fisher Scientific, Waltham, MA, USA).

The cycling conditions were as follows: an initial step of 10 min at 95 °C, 30 cycles of 30 s at 95 °C, 1 min at 55 °C (*FCGR2B* primers), 50 °C (*FCGR2A* primers) or 53 °C (*FCGR3A* primers), 1 min at 72 °C and a final extension of 7 min at 72 °C. The amplified DNA fragments of 231 bp *(FCGR2A*), 350 bp (*FCGR2B*) and 152 bp (*FCGR3A*) were visualized in a 2% agarose gel containing Midori Green (Nippon Genetics Europe GmbH, Düren, Germany) staining. PCR products were purified and sequenced using the BigDye Terminators v3.1 Ready Reaction Cycle Sequencing kit (Thermo Fisher Scientific, Waltham, MA, USA). Sequences were run on ABI 3500 capillary sequencer (Applied Biosystems, Thermo Fisher Scientific, Waltham, MA, USA), and analyzed using SeqScape version 2.7 (Applied Biosystems, Thermo Fisher Scientific, Waltham, MA, USA).

### 2.4. NA1 and NA2 Allotyping of FCGR3B

The NA1 and NA2 allotypes were amplified using forward allotype specific primers, 5′-CAGTGGTTTCACAATGAGAA-3′ for NA1 and 5′-CCATGGTACAGCGTGCTT-3′ for NA2 and a (reverse) common primer 5′-ATGGACTTCTAGCTGCAC-3′ [31]. PCRs were run as previously described. The cycling conditions were as follows: an initial step of 10 min at 95 °C, 35 cycles of 30 s at 95 °C, 1 min at 50 °C (NA1 primers) or 55 °C (NA2 primers), 1 min at 72 °C and a final extension of 7 min at 72 °C. The amplified DNA fragments of 141 bp (NA1) and 219 bp (NA2) were separated on a 2% agarose gel and visualized by Midori Green staining (Nippon Genetics Europe GmbH, Düren, Germany).

### 2.5. Haplotype Tagging and Fragment Analysis of CD72

The 13 nucleotides repeat in the intron 8 used as the haplotype tag, was studied using simple sequence repeat-PCR using specific primers [32], 6-Fam-5′-TTGGTAAGAGTGAGGGATGG-3′ (forward) and 5′-TACAAGTTTTCTCTCGGGCC-3′ (reverse). PCRs were run in 50 µL containing 100 ng of DNA, 2.5 mM of MgCl_2_, 200 µM of dNTPs, 0.4 µM of each primer and 1.25 unit of AmpliTaq Gold DNA 360 Polymerase (Applied Biosystems, Thermo Fisher Scientific, Waltham, MA, USA). The cycling conditions were as follows: an initial step at 95 °C for 10 min, 40 cycles of 30 s at 95 °C, 1 min at 55 °C, 1 min at 72 °C and a final extension of 7 min at 72 °C. PCR products were separated on a 2% agarose gel and visualized by Midori Green (Nippon Genetics Europe GmbH, Düren, Germany) staining. PCR products were diluted (1/10, 1/100). From these dilutions, 0.5 µL was combined with 9 µL of HiDi^TM^formamide (Applied Biosystems, Thermo Fisher Scientific, Waltham, MA, USA) and 0.5 µL of internal sizer GeneScan^TM^ 400HD Rox^TM^ DYE Size Standard (Applied Biosystems, Thermo Fisher Scientific, Waltham, MA, USA) and run on an ABI 3500 capillary sequencer (Applied Biosystems, Thermo Fisher Scientific, Waltham, MA, USA). The analysis and sizing of PCR products were performed using the GeneMapper software package version 3.5 (Applied Biosystems, Thermo Fisher Scientific, Waltham, MA, USA).

### 2.6. Statistical Analysis

All statistical analyses were performed with GraphPad PRISM 9.5 (Graphpad Prism Inc., San Diego, CA, USA). Datasets were checked for their normality using Anderson-Darling test. A χ^2^ test was used to assess if the studied groups had the same distribution in terms of gender. The mean and standard deviation of age were also calculated and compared using an ordinary one way ANOVA. χ^2^ and Fisher’s exact tests were implemented to compare genotype and allele frequencies depending on the sampling and using a 2 × 2 contingency table for allele frequencies and a 3 × 2 contingency table for genotype frequencies. When the distribution was deemed different, the odds ratio (OR) with a confidence interval (CI) of 95% was calculated for a 2 × 2 contingency table using Fisher’s exact test. The deviations in the observed genotypic distributions from the expected distributions based on Hardy–Weinberg law were tested using a χ^2^ test with 1 degree of freedom using the website “Online Calculator of Hardy–Weinberg equilibrium”. Linkage disequilibria were also estimated for the four polymorphisms in the studied population, using the Haploview software V4.2 [33]. The *FCGR* haplotype distributions were compared using a 4 × 2 χ^2^ contingency table. *p*-values less than 0.05 were deemed significant.

## 3. Results

### 3.1. Patients and Controls

In this study, 126 African Caribbean patients with SLE (116 females and 10 males) and 120 (109 females and 11 males) unrelated healthy individuals were included. The SLE patient group included 58 (46.03%) patients with LN and 48 (38.09%) with LWN (Table 1). However, 20 (15.8%) of the patients with SLE had an undetermined state regarding the nephritis involvement. The gender distribution did not show any significant differences among the groups. When we compared the means of age between the groups, i.e., healthy controls (53.9 ± 18.10), SLE (48.25 ± 15.79), LN (46.02 ± 14.39) and LWN (51.33 ± 16.13), no significant statistical differences were observed. Patients and healthy controls were matched according to sex and age and self-reported as Martinicans.

### 3.2. FCGR3A-158F and FCGR3B-NA2 Are Associated with Systemic Lupus Erythematosus in African Caribbeans

**Figure 1:**
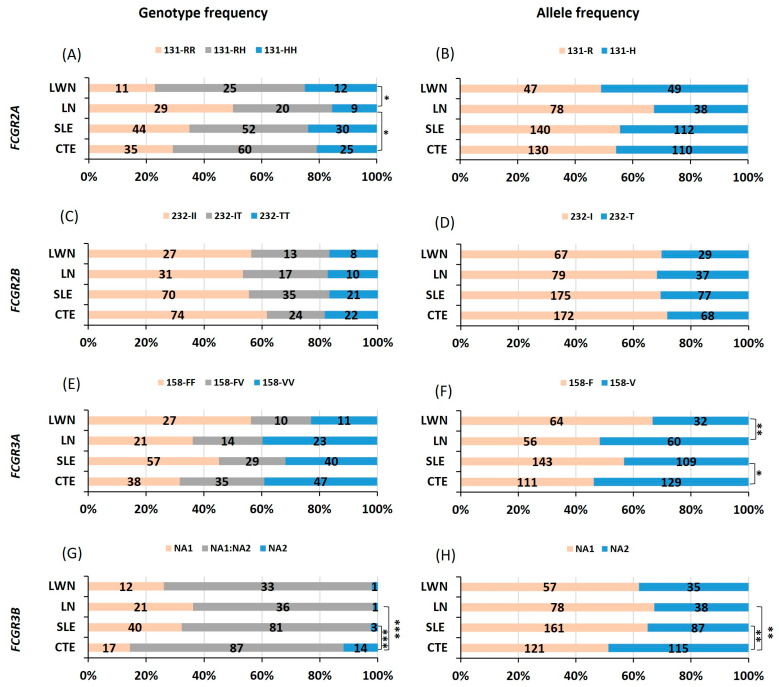
Comparison of FCGR polymorphism distributions between patients with systemic lupus erythematosus and healthy controls. Comparison of genotype and allele frequencies of FCGR2A-R131H (**A**,**B**), FCGR2B-I232T (**C**,**D**), FCGR3A-F158V (**E**,**F**) and FCGR3B-NA1/NA2 (**G**,**H**), respectively, between patients with systemic lupus erythematosus (SLE), lupus nephritis (LN), and lupus without nephritis (LWN) and healthy controls (CTE). The comparison was performed using khi² test with 2 degrees of freedom or Fisher’s exact test when the number of individuals was small. *, ** and *** indicate *p* < 0.05, *p* < 0.01 and *p* < 0.001, respectively.

We conducted an analysis of the association between *FCGR2A, FCGR2B, FCGR3A* and *FCGR3B* polymorphisms and SLE. Our results showed no differences in the genotype and allele frequencies of *FCGR2A-R131H* (Figure 1A,B) nor *FCGR2B-I232T* (Figure 1C,D) between SLE and healthy controls. In addition, the distribution of *FCGR3A-F*158V polymorphisms showed a disparity between SLE and controls (Figure 1E,F and Table A1). In fact, a higher prevalence of the 158F allele was observed in patients with SLE (57%) in comparison to controls (46%), associated with an OR of 1.52 (1.06–2.16) (Table 2). In addition, the distribution of genotype and allele frequencies of FcγRIIIB-NA1/NA2 (Figure 1G,H and Table A1) was significantly different in patients with SLE in comparison to controls (*p* = 0.0003 and 0.002, respectively). The frequency of homozygous NA1 was higher in SLE (32%) compared to controls (14%), correlating with an OR (95% CI) of 2.83 (1.49–5.38) for the homozygous NA1 in developing SLE (Table 2). When assessing allele frequencies, the NA1 allele exhibited a higher frequency in the whole group of patients (58%) in comparison to controls (50%) (Figure 1H and Table A1). This high frequency was associated with an odds ratio of 1.76 (1.23–2.54) (Table 2).

### 3.3. FCGR2AH-131R Is Associated with Lupus Nephritis

We conducted a detailed analysis of the association between *FCGR2A, FCGR2B, FCGR3A* and *FCGR3B* polymorphisms and lupus nephritis. Our findings revealed a significant correlation between *FCGR2A*-131R and lupus nephritis in our African Caribbean population. Homozygous 131R and 131R allele were over-represented in patients with nephritis (50% and 67%, respectively) in comparison to controls (29% and 54.2%, respectively) (Figure 1A,B and Table A1) with a statistical significance of (*p* = 0.024 and 0.019, respectively). The ORs associated with these findings were 2.42 (1.26–4.74) for homozygous 131R and 1.73 (1.09–2.77) (Table 2) for the 131R allele, indicating an increased risk of developing nephritis (Table 2). A further analysis within patients with LN underlined a significant difference in the distribution of the *FCGR2A*-R131H when compared to those without nephritis. The 131 R homozygous and allele were not only highly prevalent in patients with LN but also associated with higher ORs 3.36 (1.41–7.65) and 1.79 (1.22–3.65), respectively (Table 2). Additionally, the *FCGR3A-158V* allele exhibited a higher frequency (*p* = 0.007) in patients with LN (52%) compared to patients without nephritis (33.3%) (Figure 1E,F and Table A1). This frequency is associated with a risk of 2.14 (1.22–3.69) (Table 2) for developing nephritis in patients with SLE. However, no significant differences were observed in *FCGR2B-I232T* (Figure 1C,D and Table A1) nor *FCGR3B-NA1/NA2* (Figure 1G,H and Table A1) between patients with LN and controls or patients with LWN.

### 3.4. Linkage Disequilibrium at the FCGR Locus and Association with High-Risk Haplotypes

**Figure 2:**
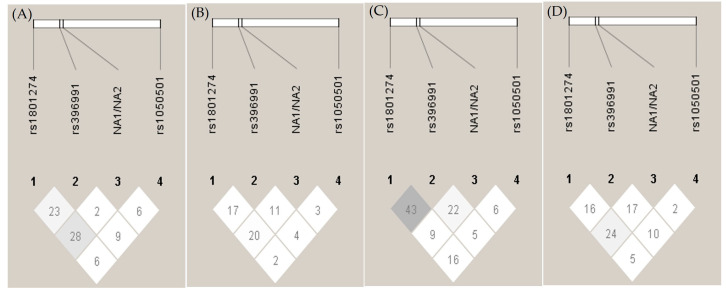
Linkage disequilibrium (LD) plot of *FCGRs*. Genetic pair-wise D’ values observed in *FCGRs*, i.e., *FCGR2A-R232H* (rs1801274), *FCGR3A-F158V (rs396991*), *FCGR3B-NA1/NA2* and *FCGR2B-I232T* (rs1050501), are shown within the diagonal boxes: (**A**) healthy control, (**B**) systemic lupus erythematosus (SLE), (**C**) SLE with nephritis, (**D**) SLE without nephritis.

Next, we studied the linkage disequilibrium (LD) of *FCGR2A*, *FCGR2B*, *FCGR3A* and *FCGR3B* in our population. The comparison between the percentages of observed heterozygous and theoretical ones showed that except for *FCGR2A*, which was consistent with the Hardy–Weinberg Equilibrium (HWE) (*p* > 0.05) in all groups, the other genes were not consistent with the HWE in any group. The association among the four studied polymorphisms was assessed through the evaluation of the linkage disequilibrium. As shown in Figure 2, D’ values suggest a weak but significant linkage disequilibrium between *FCGR2A* and *FCGR3B* in all groups except for the LN group. However, a stronger coinheritance was observed between *FCGR2A* and *FCGR3A* in the LN group (Figure 2C), associated with a high 131R-158V haplotype in patients with LN in comparison to LWN (30.4% vs. 15.8%, respectively) (*p*-value = 0.008) (Table A2) and a high OR of 2.32 (1.11, 4.87).

### 3.5. CD72-Hap2 Is Not Significantly Enhanced in FCGR2B-232T Controls

Facing the high frequency of *FCGR2B*-T232 in healthy individuals, we examined whether CD72 plays a protective role in this group. The CD72 haplotype2/2 (CD72-Hap2/Hap2) was thought to protect from SLE when associated with *FCGR2B*-232T. Using a fragment analysis, we studied the distribution of the CD72 haplotype1/2 in our population.

As shown in Table A3, a slight but not significant increase in the Hap1 genotype in healthy individuals was observed (*p* = 0.052). However, no significant differences were observed in allele and allele carrier frequencies between patients and controls.

## 4. Discussion

To date, many genetic association studies [34] and various protein-encoding genes implicated in immunity have been considered as candidate genes susceptible to SLE [35,36]. A predisposition to SLE and its potential severity have been associated with variations in *FCGRs* in several ethnic groups [29,37]. However, controversial results among ethnic groups have led to the necessity to conduct further investigations in other populations worldwide.

This study was conducted to evaluate the distribution of *FCGR2A-R131H*, *FCGR2B-I232T*, *FCGR3A-F158V* and *FCGR3B-NA1/NA2* polymorphisms in 126 patients with SLE (including 58 with LN) and 120 unrelated controls, in an indigenous African Caribbean population. Additionally, we studied the association of these polymorphisms with SLE and its severe clinical manifestation involving kidney injuries.

When *FCGR2A-R131H* was taken into consideration, surprisingly, we highlighted no association with SLE in our African Caribbean population. This result did not corroborate findings in other African and African descendant populations [38,39]. However, we identified the homozygous *FCGR2A-131R* and *FCGR2A-131R* alleles as a risk factor to develop LN in our indigenous African Caribbean population. The association of *FCGR2A*-*131R* with LN has been confirmed by meta-analysis studies [38,40], and has been observed in African Americans [41,42], Asians [31,43,44] and Latin Americans [34]. The increased risk for *FCGRIIA-131R* to develop LN could result from the lack of clearance of IgG2 containing immune complex (IC) and its deposition in organs such as kidney [45]. Interestingly, the *FCGR2A-131R* allele is associated with the poor progression of nephropathy associated with IgG deposition [27].

Regarding *FCGR3A-F158V*, the *FCGR3A-158F* homozygous and allele have been associated with SLE in various ethnic groups, including African Americans [46], Europeans [16,47], Hispanics [48]. However, discordance has been noticed in Asians [29,37,49,50,51]. On the other hand, the association of *FCGR3A*-158F with LN showed a discrepancy in several ethnic groups [52,53,54]. Our data indicates, in our African Caribbean population, an association of *FCGR3A*-158F with SLE rather than LN. Our result confirms the association of *FCGR3A-158F* with SLE in this other population of African origin and aligns with the findings in Asians where the *FCGR3A*-158F allele was significantly associated with SLE but not with LN [29,49,54]. However, when compared to LWN, the group of patients with LN exhibits a higher frequency of *FCGR3A*-158V. Thus, the aforementioned allele is described here for the first time in an African-descendent population as a high-risk factor for patients with SLE to develop LN. Our result may corroborate the finding of Alarcón et al. suggesting that the 158V homozygosity is a predictor factor of an end-stage renal disease [55]. Despite the fact that the homozygous *FCGR3A-158V* is not associated with LN in our study, we underlined a high frequency of this genotype (39%) in our patients with LN. The lack of significance may be attributed to the limited number of patients with LN in our study and needs to be confirmed in a larger LN cohort in our African Caribbean population. On the other hand, the association of *FCGR3A-158V* with LN in our study could be secondary to the observed LD between *FCGR2A* and *FCGR3A* in the LN group coupled to an increase in the 131R-158V haplotype.

FcγRIIIb-NA1/NA2 allotypes of the GPI anchored receptor have been associated with SLE. FcγRIIIb-NA2, with two potential supplementary glycosylation sites [56], has less affinity to IgG1 and IgG3 [17] and has been associated with SLE in Asians [31,49,57]. By contrast, this allele was thought to protect from SLE in Koreans [58] and was not associated with SLE in other studies [16,29,42,59,60]. In our African Caribbean population, we found an association between the homozygous FcγRIIIb-NA1 and SLE (OR = 2.829) rather than LN. However, a protective effect of FcγRIIIb-NA2 against SLE was described in our study (OR = 0.56). Additionally, the presence of zero or one copy of *FCGR3B* has been identified as a predisposing factor for developing SLE [34,61]. In this study, we found two individuals among both patients with SLE and control groups with a lack of amplification of *FCGR3B*, suggesting a FcγRIIIb deficiency in these individuals. However, it is important to note that the allotyping of *FCGR3B* was carried out based on the fragment length in electrophoresis gel separation, which did not allow the evaluation of the presence of a single copy of *FCGR3B*.

Finally, *FCGR2B-I232T* was identified as a gene candidate for susceptibility to SLE. The homozygous FcγRIIb-232T has been depicted to confer susceptibility to SLE in most Asian populations [29,31,49,62], in Caucasians [63], but not in African Americans [64]. In our study, we confirm the non-association of FcγRIIb-232T with SLE and LN in our population. This absence of association may be due to the high frequency of the FcγRIIb-232T allele (28%) in our African Caribbean population, in accordance with previous findings in African Americans [64,65,66]. FcγRIIb is the only low-affinity inhibitory receptor; The FcγRIIb-232T receptor is less efficient in translocating into lipid rafts and induces the functional impairment of the receptor [14,15]. Recently, another study revealed that FcγRIIb-232T bending toward the plasma membrane prevents the receptor to bind to the ligand [67]. Facing this high frequency in our African Caribbean population and the functional importance of this receptor, we aimed at understanding whether the non SLE African Caribbean control group has a protective factor against *FCGR2B-232T*. To do so, we studied haplotypes 1/2 of CD72 in our African Caribbean population. Haplotype 2 has been described as decreasing the risk of SLE conferred by homozygous 232T [19]. This protective effect is related to an increase in an the alternative splicing of CD72 provoking apoptosis in B cells. Additionally, the association of homozygous FcγRIIb-232T with SLE has been depicted only in groups carrying CD72-Hap1 [19]. In our study, the CD72 haplotype distribution was not significantly different in patients and healthy controls whatever the FcγRIIb genotype is. Thus, CD72-Hap2 is not responsible for the protective effect against SLE in control group.

*FCGR* gene SNPs have been associated with changes in resistance to diseases. *Plasmodium falciparum*, the main malaria parasite, is considered to be the most important evolutionary force. It causes the appearance of several alleles giving the host protections against endemic diseases [68,69,70]. For example, *FCGR2A*-131H and *FCGR2B*-232T alleles are associated with a protective effect against malaria and against its most deadly forms [63,71]. Thus, *FCGR2A-131H* and *FCGR2B*-*232T* alleles are more prevalent in population subjected to *Plasmodium falciparum* or descendants of an exposed population such as African Caribbeans [63,72,73,74], Africans and Asians [65,75]. Malaria may have continued to act as a selective pressure in African Caribbeans until its eradication from Martinique during the 20th century [76]. However, on this Caribbean Island, where arboviruses vectors proliferate throughout the year, other evolutionary pressures could exist. In fact, Martinique belongs to the most affected region by dengue virus worldwide [77]. Interestingly, the *FCGR2A*-131R allele has been described as having a protective effect against dengue hemorrhagic fever [78], the most severe form of dengue infection.

Our study has some limitations. Firstly, the inclusion was performed in a single center, which could introduce a severity bias. Secondly, the number of patients with LN is relatively small. To enhance statistical test significance, more patients with LN should be included. Moreover, in this study, 20 patients with SLE had an undetermined state regarding kidney injuries. Finally, in this study, we did not take into account the variations in the copy number of *FCGRs*.

In conclusion, our study confirms the association of *FCGR* polymorphisms with SLE and LN found in the few studied African descendent populations. In fact, our results confirm the homozygous *FCGR2A-131R* as a risk factor for patients with SLE to develop LN, but they did not confirm its association with SLE. The high frequency of *FCGR2B-232T* allele is also confirmed. In addition, the association of the *FCGR3A*-158F allele with SLE is confirmed. However, we have described the *FCGR3A-158V* allele, for the first time here, as a high-risk factor for patients with SLE to develop LN. This result could be secondary to LD in the LN group and needs to be confirmed in a larger sample of patients with LN. Additionally, we highlight an association of *FCGR3B-NA1* with SLE. Given the importance of FcγRs on IC clearance, further research is needed to understand their role in SLE pathogenesis. The distribution of *FCGR2A-R131H*, *FCGR2B-I232T* and *FCGR3A-F158V* polymorphisms mainly follows the same patterns as seen in other populations of African origin and may possibly be extrapolated to other populations with similar ancestries.

## Figures and Tables

**Table 1:** Characteristics of studied groups.

Groups	Age (Mean ± SD)	Male *n* (%)	Female *n* (%)
Healthy controls (*n* = 120)	53 ± 18.3	11 (9.17)	109 (90.83)
Systemic lupus erythematosus (*n* = 126)	48.25 ± 15.79	10 (7.94)	116 (92.06)
Lupus nephritis (*n* = 58)	46.02 ± 14.39	5 (8.62)	53 (91.38)
Lupus without nephritis (*n* = 48)	51.33 ± 16.13	2 (4.17)	46 (95.83)

SD (standard deviation), age and gender matching between patients and controls were assessed using a one-way ANOVA test.

**Table 2:** Odds ratio of FcγR polymorphisms to develop systemic lupus erythematosus and lupus nephritis, one of its severe subtypes.

*FCGRs*	OR (95% CI)	*p*-Value
SLE (*n* = 126) versus controls (*n* = 120)		
Homozygous *FCGR3B-NA1*	2.83 (1.49–5.38)	0.001
Homozygous *FCGR3B-NA2*	0.18 (0.05–0.64)	0.004
Allele *FCGR3A-158F*	1.52 (1.06–2.16)	0.02
Allele *FCGR3B-NA1*	1.76 (1.23–2.54)	0.003
Allele *FCGR3B-NA2*	0.56 (0.39–0.80)	0.003
LN (*n* = 58) versus controls (*n* = 120)		
Homozygous *FCGR2A-132R*	2.42 (1.26–4.74)	0.007
Allele *FCGR2A-132R*	1.73 (1.09–2.77)	0.02
LN (*n* = 58) versus LWN (*n* = 48)		
Homozygous *FCGR2A*-132R	3.36 (1.41–7.65)	0.004
Allele *FCGR2A-132R*	1.79 (1.22–3.65)	0.007
Allele *FCGR3A-158V*	2.14 (1.22–3.69)	0.008

Odds ratio (OR) with 95% confidence interval (95% CI) was calculated for systemic lupus erythematosus (SLE) and lupus nephritis (LN) against controls. The risk was also calculated for LN against patients with SLE without nephritis involvement (LWN). Fisher’s exact test was used, and a *p*-value < 0.05 was deemed as significant.

## Data Availability

Data are available from the corresponding author upon reasonable request.

## Appendix A

**Table A1:** Comparison of the FCGR polymorphism distribution between patients with systemic lupus erythematosus and healthy controls.

		Control (*n* = 120)	SLE (*n* = 126)	LN (*n* = 58)	LWN (*n* = 48)
Genotype Frequency *n* (%)					
*FCGR2A*-131	RR	35 (29)	44 (35)	29 (50)	11 (23)
	HR	60 (50)	52 (41)	20 (34)	25 (52)
	HH	25 (21)	30 (24)	9 (16)	12 (25)
*FCGR2B*-232	II	74 (62)	70 (56)	31 (53)	27 (56)
	IT	24 (20)	35 (28)	17 (29)	13 (27)
	TT	22 (18)	21 (17)	10 (17)	8 (17)
*FCGR3A*-158	FF	38 (32)	57 (45)	21 (36)	27 (56)
	FV	35 (29)	29 (23)	14 (24)	10 (21)
	VV	47 (39)	40 (32)	23 (40)	11 (23)
*FCGR3B*-	NA1/NA1	17 (14)	40 (32)	21 (36)	12 (25)
	NA1/NA2	87 (73)	81 (64)	36 (62)	33 (69)
	NA2/NA2	14 (12)	3 (2)	1 (2)	1 (2)
	Null	2 (2)	2 (2)	0 (0)	2 (4)
Allele Frequency *n* (%)					
*FCGR2A*-131	R	130 (54)	140 (56)	78 (67)	47 (49)
	H	110 (46)	112 (44)	38 (33)	49 (51)
*FCGR2B*-232	I	172 (72)	175 (69)	79 (68)	67 (70)
	T	68 (28)	77 (31)	37 (32)	29 (30)
*FCGR3A*-158	F	111 (46)	143 (57)	56 (48)	64 (67)
	V	129 (54)	109 (43)	60 (52)	32 (33)
*FCGR3B*-	NA1	121 (50)	161 (64)	78 (67)	57 (59)
	NA2	115 (48)	87 (35)	38 (33)	35 (36)
	Null	4 (2)	4 (2)	0 (0)	4 (4)
Allele Carrier Frequency *n* (%)					
*FCGR2A*-131	R	95 (53)	96 (54)	49 (63)	36 (49)
	H	85 (47)	82 (46)	29 (37)	37 (51)
*FCGR2B*-232	I	98 (68)	105 (65)	48 (64)	40 (66)
	T	46 (32)	56 (35)	27 (36)	21 (34)
*FCGR3A*-158	F	73 (47)	86 (55)	35 (49)	37 (64)
	V	82 (53)	69 (45)	37 (51)	21 (36)
*FCGR3B*-	NA1	104 (50)	121 (58)	57 (31)	45 (56)
	NA2	101 (49)	84 (41)	37 (39)	34 (42)
	Null	2 (1)	2 (1)	0 (0)	2 (2)

Comparison of genotype alleles and allele carrier frequencies of *FCGR2A-R131H*, *FCGR2B-I232T*, *FCGR3A-F158V* and *FCGR3B-NA1/NA2* between patients with systemic lupus erythematosus (SLE), lupus nephritis (LN) and lupus without nephritis (LWN) and healthy controls. Data are shown as a number (percentage) *n* (%).

**Table A2:** Comparison of the *FCGR* haplotype distribution, in linkage disequilibrium, between patients with SLE and healthy controls.

	Control (*n* = 120)	SLE (*n* = 126)	LN (*n* = 58)	LWN (*n* = 48)
*FCGR2A-3A* Haplotypes *n* (%)				
H-F	44 (19)	51 (24)	13 (13)	27 (32)
H-V	63 (28)	49 (23)	24 (24)	16 (19)
R-F	60 (26)	69 (32)	31 (32)	28 (33)
R-V	61 (27)	48 (22)	30 (31)	14 (16)
*p*-value		0.2	0.05	0.019
*p*_1_-value			0.008	
*FCGR2A-3B* Haplotypes *n* (%)				
H-NA1	75 (24)	79 (27)	28 (22)	36 (30)
H-NA2	69 (22)	57 (20)	19 (15)	27 (23)
R-NA1	80 (26)	94 (32)	49 (39)	34 (28)
R-NA2	83 (27)	60 (21)	31 (24)	23 (19)
*p*-value		0.13	0.05	0.34
*p*_1_-value			0.10	
*FCGR3A-3B* Haplotypes *n* (%)				
F-NA1	62 (24)	81 (32)	35 (30)	33 (35)
F-NA2	60 (24)	52 (21)	20 (17)	23 (25)
R-NA1	73 (29)	67 (27)	36 (31)	20 (22)
R-NA2	60 (24)	50 (20)	26 (22)	17 (18)
*p*-value		0.23	0.44	0.15
*p*_1_-value			0.25	

Comparison of *FCGR* haplotype distribution, in linkage disequilibrium, between patients with systemic lupus erythematosus (SLE), lupus nephritis (LN) and lupus without nephritis (LWN) and healthy controls. Data are shown as a number (percentage) *n* (%). *p*-value of Χ^2^ test for a 4 × 2 contingency table was calculated against controls. *p*_1_-value was calculated between LN and LWN. *p* and *p*_1_ < 0.05 were deemed significant.

**Table A3:** Haplotypes and allele frequency distribution of CD72.

	Controls (*n* = 120)	SLE (*n* = 126)	LN (*n* = 58)	LWN (*n* = 48)
Genotype Frequency *n* (%)				
Hap1/Hap1	22 (18.3)	11 (9.2)	6 (10.7)	3 (6.5)
Hap1/Hap2	51 (42.5)	67 (52.6)	30 (53.6)	24 (52.2)
Hap2/Hap2	47 (39.2)	48 (38.2)	20 (35.7)	19 (41.3)
*p*-Value		0.057	0.282	0.149
*p*_1_-Value			0.69	
Allele Frequency *n* (%)				
Hap1	95 (39.6)	89 (35.5)	42 (37.5)	30 (32.6)
Hap2	145 (60.4)	163 (64.5)	70 (62.5)	62 (67.4)
*p*-Value		0.32	0.7	0.2
*p*_1_-value			0.46	
Allele Carrier Frequency *n* (%)				
Hap 1	73 (60.8)	78 (61.8)	36 (64.3)	27 (58.7)
Hap 2	98 (81.6)	115 (91)	50 (89.3)	43 (93.4)
*p*-value		0.6	0.89	0.55
*p*_1_-value			0.67	

Comparison of the distribution of haplotype 1 and 2 (Hap1 and Hap2) of CD72 in groups of patients with systemic lupus erythematosus (SLE), lupus nephritis (LN) and SLE without nephritis (LWN) and healthy controls. *p*-value of Fisher’s exact test was calculated against healthy controls. *p*_1_-value was calculated between LN and LWN. *p* < 0.05 was deemed significant.
